# Learning predictive signatures of HLA type from T-cell repertoires

**DOI:** 10.1371/journal.pcbi.1012724

**Published:** 2025-01-06

**Authors:** María Ruiz Ortega, Mikhail V. Pogorelyy, Anastasia A. Minervina, Paul G. Thomas, Thierry Mora, Aleksandra M. Walczak

**Affiliations:** 1 Laboratoire de physique de l’École Normale Supérieure, CNRS, PSL Université, Sorbonne Université, and Université Paris-Cité, Paris, France; 2 Department of Host-Microbe Interactions, St. Jude Children’s Research Hospital, Memphis, Tennessee, United States of America; University of Pittsburgh, UNITED STATES OF AMERICA

## Abstract

T cells recognize a wide range of pathogens using surface receptors that interact directly with peptides presented on major histocompatibility complexes (MHC) encoded by the HLA loci in humans. Understanding the association between T cell receptors (TCR) and HLA alleles is an important step towards predicting TCR-antigen specificity from sequences. Here we analyze the TCR alpha and beta repertoires of large cohorts of HLA-typed donors to systematically infer such associations, by looking for overrepresentation of TCRs in individuals with a common allele.TCRs, associated with a specific HLA allele, exhibit sequence similarities that suggest prior antigen exposure. Immune repertoire sequencing has produced large numbers of datasets, however the HLA type of the corresponding donors is rarely available. Using our TCR-HLA associations, we trained a computational model to predict the HLA type of individuals from their TCR repertoire alone. We propose an iterative procedure to refine this model by using data from large cohorts of untyped individuals, by recursively typing them using the model itself. The resulting model shows good predictive performance, even for relatively rare HLA alleles.

## Author Summary

The deep sequencing of immune repertoires from blood samples promises to offer diagnostic and precision medicine tools, and to help with the analysis of treatments and vaccinations and the design of immunotherapies. This study shows how, by training models on large datasets of annotated and unannotated repertoire sequencing, the human leukocyte antigen (HLA) type of patients can be computationally determined from their T-cell repertoire. This tool could be useful for typing repertoires for which the HLA is unknown, and to gain insight into the HLA restriction of T-cell receptor epitope specificity. It also provides lists of T-cell receptors that are associated to each HLA allele.

## Introduction

T cells are one of the building blocks of adaptive immunity. They accomplish a highly precise and tailored immune response using membrane-bound antigen-specific T cell receptors (TCR), a heterodimer composed of an alpha and a beta chain. To cover all possible pathogens, T cells rely on their large TCR sequence diversity [1]: the gene of each chain undergoes somatic V(D)J recombination during T cell development, resulting in the generation of extensive genetic heterogeneity in the TCR repertoire.

The *αβ* TCR interacts with short peptide fragments displayed on the surface of antigen presenting cells by the major histocompatibility complex (MHC) molecule. In humans, these structures are encoded by the highly polymorphic human leukocyte antigen (HLA) genes. Each individual carries up to 2 alleles of each of the 3 class-I (A, B, and C) and multiple class-II (DP, DQ, and DR) MHC genes, each binding a large but restricted set of different peptides with common sequence features [2–4]. This MHC restriction introduces an extra layer of complexity to TCR specificity to particular combinations of peptides and MHC molecules [5, 6], with strong heterogeneity across individuals. Despite its importance for antigen specificity, most efforts focus on disentangling peptide specificity [7–10], while the interaction between HLA type and the TCR repertoire remains poorly understood, especially for less common HLA alleles.

Advances in high-throughput repertoire sequencing [11] have allowed for unprecedented insights into the composition of immune repertoires, which constitutes a dynamic register of the immune challenges encountered by the organism [12]. Public TCR clones, which are found in several individuals, have raised great interest as possibly playing functional roles in antigen recognition [13, 14]. Many factors affect TCR sharing, including biases in the VDJ recombination process and convergent recombination [15–19], thymic and peripheral selection [20], as well as shared diseases and HLA background [12, 21–23].

Here we analyze the TCR repertoire of 1039 HLA-typed human donors from three different cohorts. We follow and expand on prevoius approaches [12, 21, 24] to identify public TCR sequences that are enriched in individuals with a given HLA allele. We use those sequences to build a reliable predictor of HLA type from repertoire data. Our analysis combines alpha and beta chain information, and makes predictions for both class-I and II HLA alleles, leading to more accurate and comprehensive predictions than previous approaches, by leveraging larger datasets and accounting for 4-digit resolution of HLA alleles. Our HLA type predictor is encoded in new software package, HLAGuessr, available at https://github.com/statbiophys/HLAGuessr. Finally, we demonstrate that our method can be used to discover new HLA-TCR associations from the repertoires of *untyped* individuals based on our predictor, opening up the possibility to aggregate data from a large body of datasets for which the conventional HLA typing is unavailable.

## Results

### Disovery of HLA-associated TCRs

We first sought to identify lists of TCRs associated with each HLA allele, using an aggregate dataset of TCR repertoires of 1039 HLA-typed donors from three different sources comprising: the *Russell et al.* dataset with 718, 000 unique TCR*α* and 1, 016, 000 unique TCR*β* sequences from 237 donors [25], the *Emerson et al.* dataset with more than 7 × 10^7^ TCR *β* unique sequences from 610 donors [21] and the *Rosati et al.* dataset with 7 × 10^6^ TCR*α* and 1 × 10^7^ TCR*β* sequences from 192 healthy and Crohn’s disease patients [26]. We only considered TCR sequences that were seen in at least 3 people across the cohorts, leaving us with 654,535 alpha chains, and 4,927,204 beta chains to analyse. For each individual, the HLA type was encoded in a list of up to 6 HLA type I alleles (2 of each of A, B, C) and at least 10 for HLA type II (4 possible isoforms of DP, 4 of DQ and 2 of DR1, while DR3–5 have variable presence in humans). There were 316 HLA alleles in total.

**Fig 1:**
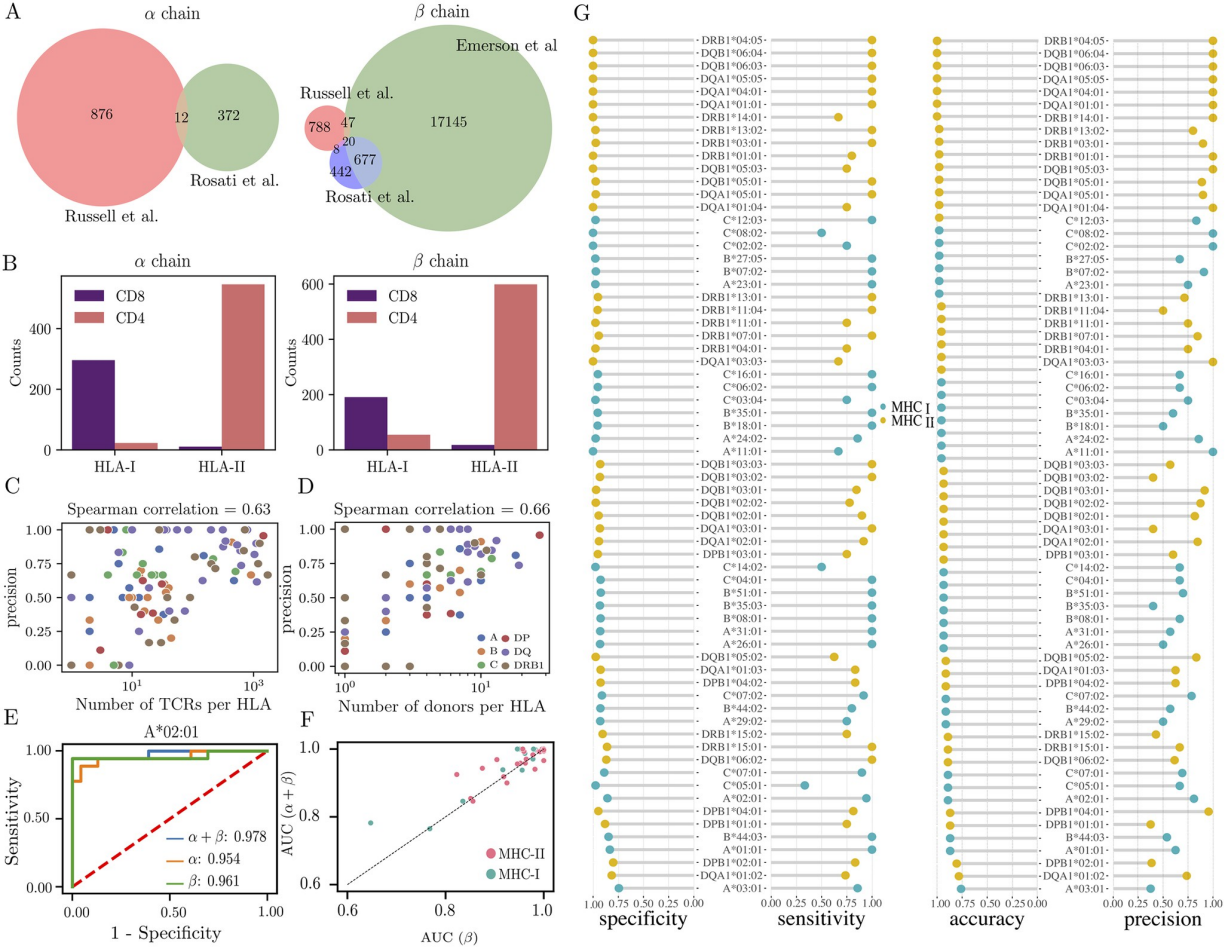
Summary of HLA predictor performance. (A) Venn diagram of the alpha and beta chains significantly associated with an HLA allele after performing the Fisher’s exact test in our three different cohorts of data. Overlap corresponds to sequences associated to the same HLA in the different datasets. (B) The number of HLA-associated TCRs show concordance between their CD4+ and CD8+ phenotype and the MHC class of the HLA allele. (C-D) Correlation between the precision of the classifier with (C) the number of found associations and (D) the frequency of the HLA allele in the cohort. Dots are color-coded to identify the corresponding HLA locus. (E) Receiving Operator Characteristic (ROC) of the classifier for the A*02:01 allele in the validation data set, using either each chain separately or together. (F) Comparison of the area under the curve (AUC) of the ROC for each of the tested alleles when training the model only with beta chain (x axis) vs. with beta + alpha chain (y axis). The performance reaches its best when using both chains in 26 out of 47 alleles. (G) Performance metrics of the HLA classifier on the validation dataset. Specificity = TN/(TN+FP), Sensitivity = TP/(TP+FN), Accuracy = TP+TN/(TP+TN+FP+FN), Precision = TP/(TP+FP), where T/F stand for true/false, and P/N for positives/negatives.

For each TCR sequence (alpha or beta), and each HLA allele, we performed Fisher’s two-tailed exact test to ask whether the presence of that TCR in individuals was associated with the presence of that allele (see Methods for details) in each cohort. To control for multiple testing, we set the p-value threshold for a significant association using the Benjamini-Hochberg procedure, so that the false positive rate is 1%. This procedure yields a list of TCR*α* and TCR*β* sequences that are associated with a particular HLA allele, with a level of significance indicated by the p-value (see S1 Fig and S1 Table). The results for each of the three cohorts analysed separately are summarized in the Venn diagrams of Fig 1A, where the overlap refers to TCR clonotypes that were associated with the same HLA in different cohorts. We found associations in the *Russell et al.* cohort between 894 TCR*α* with 51 HLA alleles, and between 863 TCR*β* and 58 HLA alleles. In the *Emerson et al.* cohort, we report 17889 TCR*β* associated to 116 HLA alleles; this number broadly agree with the previous analysis of [12] with minor discrepancies due to a different definition of alleles: we restrict our analysis to single HLA alleles, while [12] also considered haplotypes or co-occurence of several alleles. In the *Rosati et al.* cohort, we find 384 TCR*α* associated with 48 HLA alleles, and 1147 TCR*β* associated to 58 HLA alleles. The number of significant associations is largely determined by the number of sequences in each dataset, which explains large differences between the cohorts.

As a first check of the consistency of the discovered associations, we verified that the CD4/CD8 phenotype of each TCR reported in the *Russell et al.* dataset (as determined by flow cytometry) matched their associated HLA class II and class I alleles. CD4^+^ cells recognize antigens presented on class II MHC molecules (encoded by the DP, DQ, and DR HLA genes), while CD8^+^ respond to antigens presented on class I MHC molecules (encoded in the A, B, and C HLA genes) [27]. Fig 1B shows the number of CD4 and CD8 TCR *α* and *β* sequences that were associated to any HLA of class I or II. As expected, the majority of CD8^+^ TCRs are associated to HLA alleles of class I, and the majority of CD4^+^ TCRs to HLA alleles of class II. Exceptions to this rule are not necessarily all due to noise, since some sequences are simultaneously marked as CD4 and CD8 cells, probably because the same alpha or beta chain may be used in two distinct T cell clones with different HLA specificities. We find more HLA-II associated clones, in agreement with previous studies [12]. A likely explanation for this is the overall higher abundance of CD4+ T cells in peripheral blood, which biases the results towards a higher number of HLA-II-associated clones.

### HLA-associated TCR predict the HLA type

To maximize statistical power, we repeated the association discovery procedure on a random 70% training portion of the merged dataset combining all three cohorts, resulting in a list of 2570 TCR*α* and 63060 TCR*β* sequences significantly associated to at least one HLA allele. This number is much larger than the sum of the numbers of associations from each cohort taken separately. For each HLA allele, we fit a logistic regression model on the training set predicting whether a given donor has this allele or not, depending on whether it has or not each TCR sequence (both alpha and beta) found to be associated to that allele. We excluded from this fit sequences from the *Emerson et al.* dataset whenever the alpha chains were used. Regression was done with an L1 regularization, which strength was tuned to optimize the accuracy of the prediction on the testing set made of the remaining 30% portion of the data (see details in Methods).

Model predictions were then evaluated on an independent dataset of 46 HLA-typed individuals from *Milighetti et al* [28]. Four measures of performance for each analysed HLA allele are reported in Fig 1G: accuracy (proportion of individuals for which the prediction is correct), precision (proportion of true positives among positive predictions), specificity (propotion of people without the HLA allele who are correctly predicted), and sensitivity (proportion of people with the HLA allele who are correctly predicted). The accuracy and specificity are good for almost all alleles, due to the fact that the prevalance of each HLA allele is relatively low in the population, meaning that there many more negative than positive examples. We expect that HLA alleles with lower prevalence are harder to predict [29–31]. Indeed, precision is correlated with both the total number of TCR associated to the HLA allele (Pearson *ρ* = 0.63, *p* = 6 × 10^−14^, Fig 1C), and the prevalence of that HLA allele in the cohort (Pearson *ρ* = 0.66, *p* = 1.2 × 10^−15^, Fig 1D). A more complete way to report the performance of the classifier is to plot the Receiver Operating Characteristic (ROC) curve, which shows sensitivity as a function of specificity as one varies the threshold on the logistic regression score (this threshold was set earlier by the logistic regression to maximize cross-entropy, see Methods). Example ROC curves are shown in Fig 1E for HLA A*02:01, using either both alpha and beta chains, or each separately. The intrinsic imbalance of the classification problem, where most individuals are negative for any given HLA allele, suggests that other metrics than the ROC may be more appropriate to quantify the accuracy of the prediction, such as the precision-recall curve, which is often used for such imbalanced tasks. Using that metric instead of the ROC gives similar results (S2A Fig).

The ROC curve may be conveniently summarized in a threshold-independent manner by the area under the curve (AUC), which is 1 if the predictor is perfect, and 0.5 if it is random. Fig 1F shows the AUC of all HLA alleles, comparing predictors using only beta chain or both of them together. Although TCR*α* and TCR*β* could be used for HLA typing independently, the alpha chain predictive power is overall lower (AUC = 0.84 ± 0.13, *p* = 8 × 10^−9^, Wilcoxon test) than beta chain (AUC = 0.95 ± 0.07). Using both chains together confer a slight advantage compared to the beta chain alone (AUC = 0.97 ± 0.05, *p* = 0.02), confirming that both chains have information about the HLA restriction (S2B Fig and S2 Table). This is further confirmed with the outlier shown in Fig 1F, corresponding to the allele B*32:01, which is detected using only alpha chains.

**Fig 2:**
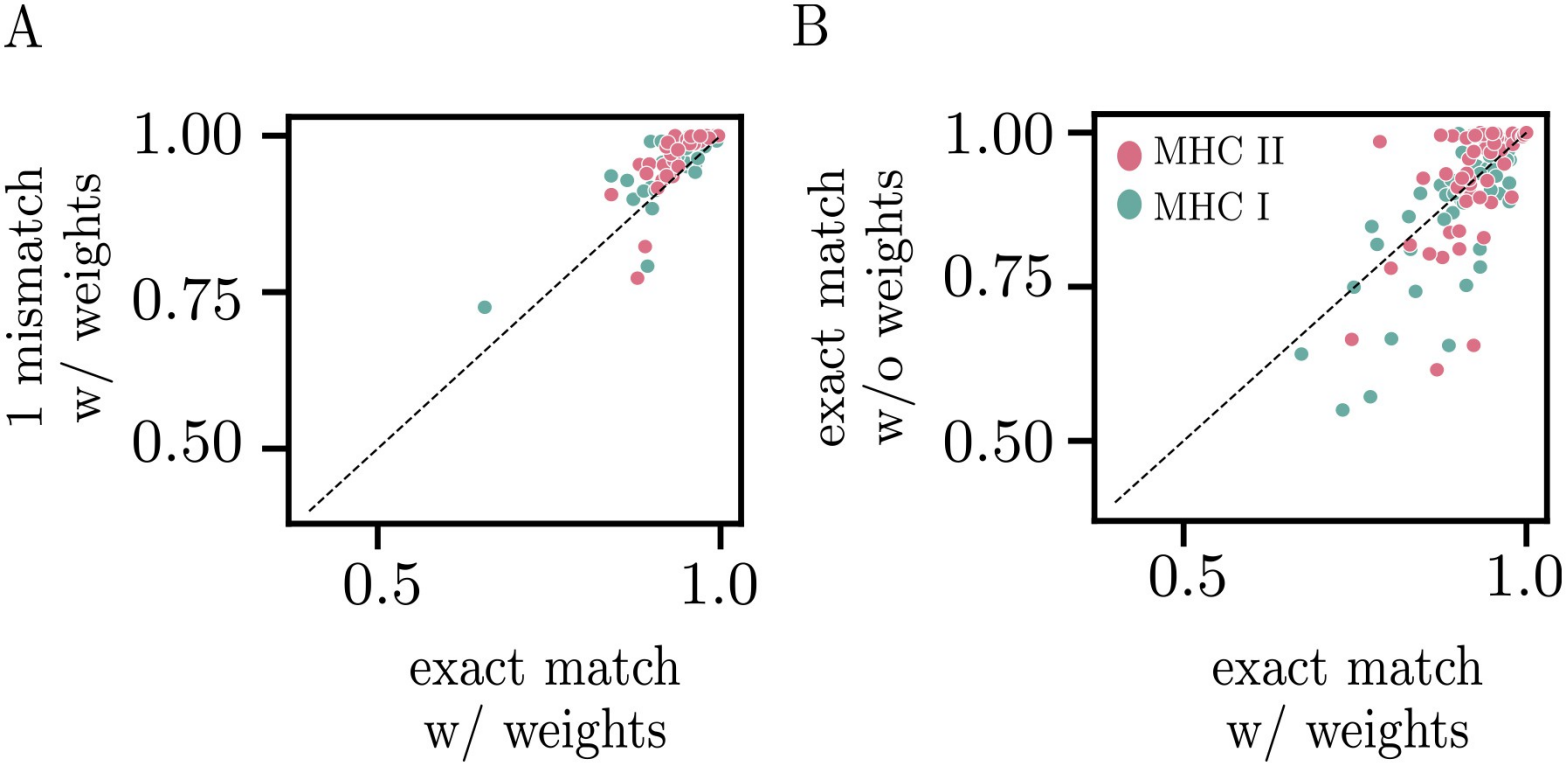
Algorithm variants. (A) Comparison of AUCs between the basic classifier (based on exact matches with HLA-associated TCR), and a classifier where single amino acid variants are counted as a match. (B) Comparison of AUCs between the basic classifier, where the weights associated to each HLA-associated TCR are learned using logistic regression, and a simpler classifier that simply counts the number of matches (without weights, i.e. all weights = 1), which was used in [21].

### Fuzzy TCR sequence matching improves HLA-typing

We also tested two variants of the algorithm. Since TCR specific to the same target often have similar sequences [21, 32–35], one could extend the search for the presence of HLA-associated TCRs to their neighbors in sequence space. We re-trained the logistic regressor by counting each HLA-associated sequence as present if either that exact sequence or a variant with one amino acid mismatch in CDR3 was found in the repertoire. Fig 2A shows that this more liberal way to include HLA-associated sequences leads to increased performance on average, as measured by the AUC (0.95 ± 0.05, *p* = 5 × 10^−8^ Wilcoxon test). While this inexact matching improves HLA prediction quality, it is more computationally expensive. For this reason, we used the basic classification algorithm in further analysis.

In the second variant, which is equivalent to the original method of Ref. [21] and requires no logistic regression, the prediction of whether the individual is positive or negative for an HLA is just determined by the total number of HLA-associated sequences found in their repertoire. Fig 2B shows this simpler strategy offers a similar quality of prediction (AUC = 0.91 ± 0.10, *p* = 0.96 Wilcoxon test) to logistic regression. This is probably due to the type of penalty used for the inference of our model (L1), which encourages sparse solutions as the penalty becomes more stringent. S3 Table contains a more direct comparison to the results of Ref. [21], as well as Ref. [24], which uses the same idea but using TCR associated to HLA by an approximate *χ*^2^ test instead of Fisher’s exact test. In those original work prediction was made on 2-digit HLA instead of 4 digits as we do in this work, which yields higher performances at the expense of HLA typing resolution. We also trained a classifier using an L2 penalty to quantify the different contributions from distinct TCRs to the prediction score (S3 Fig). However, the L1 penalty resulted in a better sensitivity, and so we used L1 for further analysis.

### Features of HLA-associated TCRs

**Fig 3:**
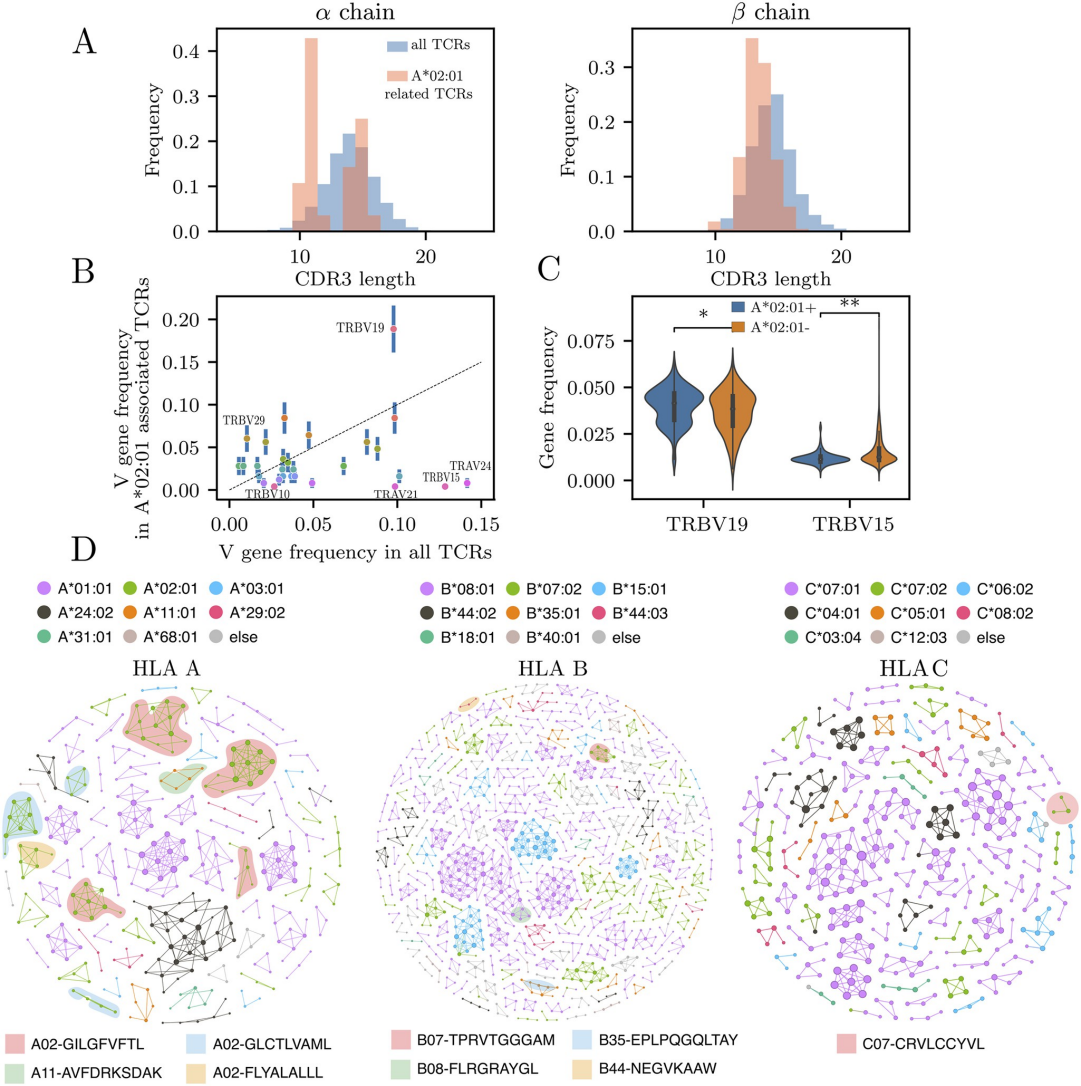
Features of the HLA-related sequences. (A) CDR3 length distribution of A*02:01-associated TCRs, compared to all TCRs. (B) V gene frequency usage comparison between the same two subsets. Some V gene families are preferentially used in A*02:01-related TCRs (TRBV10, TRBV19, TRBV29) while other genes are underrepresented (TRBV15,TRAV24,TRAV21). (C) Whole-repertoire frequencies of two representative differentially used genes, TRBV19 and TRBV15, in A*02:01 positive vs negative individuals show small but statistcally significant difference in those genes. (D) Network analysis of TCR*β* chains associated with different A, B and C HLA-alleles (TCR*α* were excluded since they formed negligible networks). Each node represent an amino acid CDR3 + V gene clonotype. Edges connect clonotypes that have at most on amino acid mismatch but the same length and V gene. Each color represent the specific HLA to which the TCR is responsive. Shadow color correspond to epitopes for which those TCRs are found to be responsive from the VDJdb database [37].

We then asked whether HLA-associated TCRs share some common features, focusing on HLA A*02:01, the most abundant HLA allele in our dataset. Fig 3A shows the CDR3 amino acid length distribution for both alpha and beta chain (28 and 221 sequences, respectively). The CDR3-length distribution of the beta chain of HLA A*02:01-associated sequences seems to be heavily skewed towards shorter sequences. *α* chains strongly deviates as well when compared with random TCRs (Fig 3A). Since TCR:pMHC binding takes place through the V gene-encoded CDR1 and CDR2 contacting the conserved helical residues of the MHC [36], we expect that some conserved structural footprint might be observed in V gene usage. A*02:01-associated TCRs showed a biased usage of V genes relative to generic TCRs, with frequency enrichment in TRBV10 (0.03 vs 0.006), TRBV19 (0.2 vs 0.1), TRBV29 (0.06 vs 0.01), TRBV5 (0.03 vs 0.008), and under-representation of TRBV15, TRAV24, TRAV5 V gene families (see Fig 3B). We reasoned that the usage of those genes may be biased in the whole repertoire (not restricted to the HLA-associated TCR) of A*02:01 positive individuals as well. We found significant differences between the usage of TRBV19 and TRBV15 (the two genes with the largest deviation) of A*02:01 positive versus negative individuals (*p* = 1 × 10^−4^ and *p* = 6 × 10^−17^, Mann-Whitney U test, see Fig 3C). This suggests that A*02:01-associated TCRs biases towards certain V genes could affect the repertoire of A*02:01 positive people as a whole. However, while V gene usage biases might be influenced by the MHC molecule, it is more likely that they arise from selection by specific epitopes within the context of an immune response. Certain epitopes might preferentially engage TCRs with particular V genes, leading to an enrichment of these TCRs in the repertoire. Ref. [12] reported that some of the most strongly A*02:01-correlated TCRs using TRBV19 and TRBV5 were found to be positive for two viral epitopes, specifically influenza M1_58_ and Epstein-Barr virus BMLF1_280_ [33]. This suggests that the V gene bias may be partly driven by particular epitopes rather by direct binding to MHC.

Next, we investigated if similar TCRs are associated with the same HLA alleles, as already suggested by the improved performance of the mismatch-tolerant version of the HLA-type predictor (Fig 2A), where similar TCR to the ones actually associated to the HLA also help determine if an individual is positive for that HLA.

In Fig 3D, we represent HLA class I-associated TCRs as similarity networks, where nodes represent TCR clonotypes, defined by the CDR3 sequence and the V gene family, and edges are drawn between two sequences of the same length that differ by at most one amino acid. Each HLA allele may be represented by several clusters, corresponding to distinct specificity classes within the same HLA, and some of these specificity classes can be mapped out onto specific antigens by matching clonotypes from the VDJdb database [37]. We downladed human TCRs from VDJdb (accessed on 22 September 2023), and excluded sequences from the 10x Genomics highly multiplexed dextramer dataset [38]. We then searched for exact matches of both CDR3 amino acid sequence and V gene family. Almost all clusters associate with a single HLA allele, with no mixing (Fig 3D). This suggests that during thymic selection and further clonal expansion driven by antigen recognition each HLA selects exclusive clusters of closely related TCR sequences.

The number of memory T cells progressively increases with age due to antigen exposure [39, 40], and we expect the repertoire to focus on specific HLA types during that process. We asked how the number of HLA-related TCRs in each individual from *Britanova et al.* changes with age. We found that the relative number of HLA-related TCRs found in repertoires from individuals from 0 to more than 100 years old shows a weak yet significant correlation with age (*r* = 0.29, *p* = 0.01, S4 Fig).

### Predicting HLA alleles in untyped repertoires

Our HLA predictor was trained on the repertoires of individuals whose HLA type was known. However, there exist many more datasets for which this information is absent. We asked if we could leverage this large amount of untyped repertoire data to improve the performance of the classifier in an iterative way, by first typing untyped repertoires computationally using our logistic predictor, and then use these repertoires to discover new TCR-HLA associations. To demonstrate the feasibility of this approach, we used two additional TCR*β* repertoire datasets: one comprising 108 healthy individuals from Ref. [41], and the second including 1414 donors with a confirmed SARS-CoV-2 infection at various timepoints following the peak of the disease, totaling 1.6 × 10^8^ reads [42], and focused on 4 carefully selected HLA alleles. We picked A*02:01, DPB1*04:01 and DQB1*01:02 because of their high prevalence (around 50%) in the population, which allows for more robust statistics. The initial classifier showed only moderate accuracy for DPB1*04:01 and DQB1*01:02 despite their abundance, suggesting room for improvement. We chose the 4th allele DPB1*02:01 because of its mediocre performance in the initial classifier (AUC of 0.77), again to test the potential for improvement of an iterative approach.

We divided the cohort of untyped individuals into random subgroups of 100. We then applied the following recursion to each of the 4 HLA alleles. We first used the classifier previously trained on the typed cohort to predict the HLA positive or negative status of the first subgroup of 100. We then added this newly typed subgroup to the initial cohort, and re-ran the Fisher’s exact test to obtain a new list of HLA-associated TCR sequences. This updated list of HLA-associated sequences is expected to be longer than the previous one, as it is based on a larger cohort. We use this list to re-train a logistic regression classifier on the augmented cohort (initial cohort plus the 100 subgroup), using again a 70%/30% split between training and testing to optimize the regularization parameter. The new classifier is used to type the next subgroup of 100 individuals. The procedure is repeated until all subgroups have been typed.

**Fig 4:**
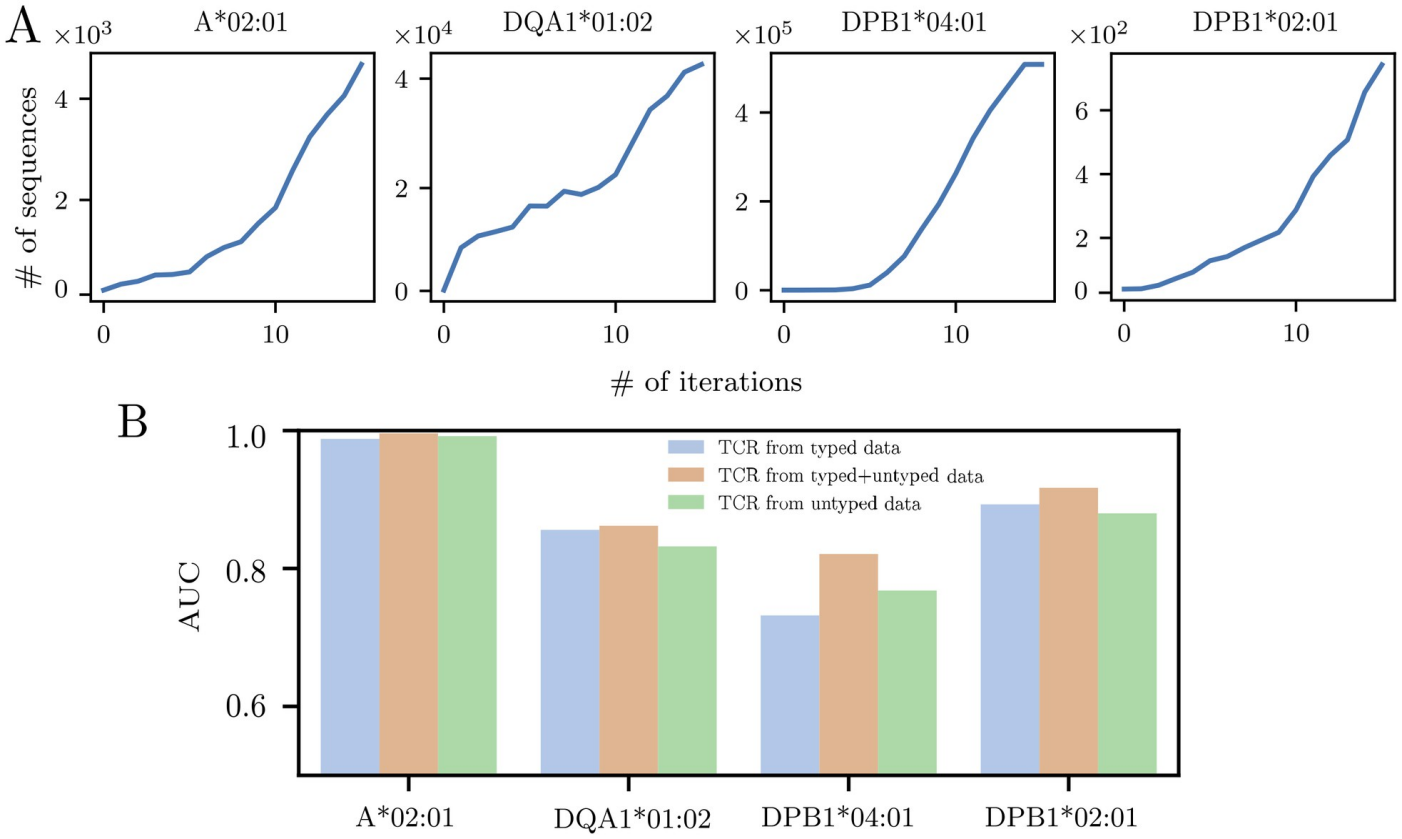
Iterative HLA-typing process. (a) Number TCRs found to be significantly related to the HLA alleles A*02:01, DQA1*01:02, DPB1*04:01, DPB1*02:01 after recursively applying our HLA predictor in two untyped datasets [41, 42]. In this procedure, donors are iteratively typed by groups of 100 patients. The HLA related sequences found in our newly typed subcohort are used to infer the HLA type of the next 100 patients. (b) Comparison of the AUC when predicting in the validation dataset using the sequences inferred in the first round from our initial typed cohort (blue bar) the ones learned exclusively from the untyped cohorts after 14 iterations (green bar) or the combination of both subcohorts (orange bar). The performance is shown to be maximized when using all the available HLA-related TCRs, supporting the quality of the information acquired through this iterative process.

With each iteration and the inclusion of more individuals, the statistical power is increased, leading to an exploding number of HLA-associated TCR, notably after the 10th iteration (see Fig 4A). However, since there are errors in the computational typing, one might worry that these errors get amplified and propagated through the iterations. In order to mitigate this effect and also to avoid overfitting due to a very large number of associated sequences, we tune the threshold on the p-value of the Fisher’s exact test so that the number of HLA-associated sequences is proportional to the number of included donors, with a ratio fixed to its value found in the initial cohort.

Fig 4B shows that the performance (AUC) of the classifier on the validation cohort is improved in the iterative procedure (orange) compared to the original classifier (blue), suggesting that the new associations provide additional information. To further validate the new associations, we trained a third logistic classifier using the presence or absence of the novel HLA-associated TCR only, i.e. sequences that were not present in the original list. This classifier yielded comparable and sometimes even better performance (in green) than the original classifier, despite being based on sequences that were identified using untyped cohorts. This demonstrates the potential of the iterative method to uncover novel associations with strong predictive power.

## Discussion

Despite the advances in the high-throughput sequencing technology of immune repertoire made over the last decade, decoding the individual’s past and present immunological status from TCR repertoire remains a formidable challenge. This is mainly due to the lack of tools to decipher T cell specificity just from the T cell receptor amino acid sequence. This problem involves three components—TCR, peptide, MHC. While the peptide–MHC binding can now be well predicted [43], progress on TCR–antigen binding prediction has been hindered by lack of data, despite some recent progress on the computational side [44–46]. However, direct association between the MHC and the TCR has not been much studied, although recent work has shown that HLA association could be predicted from the sequence features of the TCR using machine learning methods [47, 48]. Our results shed light on this problem by deciphering the HLA restriction of public TCRs found in the repertoires of large cohorts of donors.

Our method is based on a combination of a statistical test for HLA-TCR association, and a classifier for predicting the presence of absence of HLA, originally introduced in Ref. [21] for beta chains. The main reason for our improved preformance is the incorporation of a larger dataset, including alpha as well as beta chains. In addition, we provide predictions with up to 4-digit resolution. Another key contribution of this paper is the iterative “bootstrapping” approach, which allowed us to extract HLA association information from repertoires that were untyped, by leveraging our computational typing predictions.

An interesting question is whether TCRs with the same HLA restriction share sequence features that would be predictive of MHC binding. Our network analysis carried out through similarity clusters confirms previous intuition [49, 50] that TCRs having similar protein sequences are likely to be specific to the same HLA allele, and we could match some of those clusters to particular viral epitopes. These observations are consistent with reports that HLA restriction is predictable from the TCR sequence alone [47, 48], which could in turn help refine models of TCR specificity, by accounting for HLA restriction.

Our analysis combines deep bulk *α* and *β* repertoires, allowing us to explore how the alpha chain influences the HLA preference of the TCRs. For some HLA alleles (e.g. B*15:18, DRB3*01:01, DRB4*01:03 or DRB5*01:01), most of the associations we found correspond to alpha clonotypes. However, the *β* chain alone was sufficient to type donors computationally, with little or no improvement upon adding *α* chain information. We expect HLA restriction to be determined by the combination of both chains. Since the repertoire data we used was unpaired, we cannot exclude the possibility that HLA associations learned from paired data could have more predictive power. However, such datasets (deep, chain-paired repertoires of large cohorts of typed individuals) do not exist to the best of our knowledge.

Because the predictive power of our method depends crucially on the number of donors, especially in the HLA-TCR association discovery step, it is limited to common HLA types. Some HLA types are genetically linked, meaning that they tend to occur together. A more general statistical framework could leverage these correlations to improve prediction. Another way to improve statistical power could be to relax the notion of publicness used to allow for imperfect matches (e.g. 1 amino acid different). We showed that this helped the logistic classifier step. Applying this idea to the association discovery step is less straightforward as it would require generalizing the Fisher’s exact test, but it could help increase number of found associations, potentially boosting the predictive power of the classifier.

Our algorithm ignores constraints and biases imposed by the genome. For instance, individuals can only harbor 2 distinct alleles for each locus. This could be imposed *a posteriori* in our method by selecting the best 2 predicted ones. In addition, some loci are genetically linked (e.g. B and C), implying priors about what combination of HLA alleles is expected to be found in a given haplotype. Integrating these effects with appropriate controls for unintended biases could help further improve HLA prediction.

In addition to our core method, we presented an iterative way to exploit information from untyped repertoires to find new HLA-TCR associations. As the number of publicly available repertoires increases, often from donors for which the HLA type is unreported, this method could prove useful to leverage future datasets to populate expanded lists of HLA-associated TCRs, and to improve the predictions of computational HLA-typing.

## Methods

### Datasets

The repertoires initially used for the training of the HLA classifier were extracted from three different datasets described in the main text. The *Russell et al.* dataset [25] is available in The BioProject database under accession code PRJNA762269. The *Emerson et al.* dataset [21] is available at https://clients.adaptivebiotech.com/pub/Emerson-2017-NatGen. The *Rosati et al.* TCR dataset [26] is available on the ENA database with study accession number PRJEB50045, while HLA genetic data was obtained from the authors upon request. The *Milighetti et al.* validation dataset [28] is available at https://github.com/innate2adaptive/CovidsortiumTCRExpanded. All subjects included for the learning phase were typed at 4 digits resolution. For the iterative procedure, the first dataset [41] was downloaded from the NCBI SRA archive using the Bioproject accession PRJNA316572 [41] and the second [42] with donors following a confirmed SARS-CoV-2 infection [42]. From every dataset we excluded donors with incomplete HLA-typing results (i.e. not all loci were known) and duplicated repertoire samples (i.e. coming from different time points) from the same donor Raw sequencing reads were then aligned against a reference database of human V-, D- and J- segments using the MiXCR pipeline [51]. The resulting T cell receptor CDR3 repertoires were further filtered to remove out-of-frame and stop codon-containing CDR3 variants.

### HLA prediction

From the list of HLA-related TCRs, we trained a logistic regressor to predict a given HLA phenotype of an individual from TCR sequencing data.

This input format allows the model to learn and assign different weights to each sequence, maximizing those that are crucial for identifying an allele while minimizing the possible noise introduced by incorrectly identified alleles.

In the logistic model, the probability for a donor to be positive for a particular HLA allele *a* reads:

$$\begin{array}{c} p_{a}=\frac{1}{1+e^{-w_{a1}x_{1}-\ldots -w_{an}x_{n}-v_{a}y}}, \end{array}$$ (1)

where *x*_1_, …, *x*_*n*_ denote the absence (*x*_*i*_ = 0) or presence (*x*_1_ = 1) of each HLA-associated TCR indexed by *i* in the individual. *w*_*ai*_ are TCR- and HLA-specific weights learned during training. *v*_*a*_ is an extra learnable parameter to encode the information that the alpha chain was sequenced (*y* = 1) or not (*y* = 0) in that individual.

A model was separately trained for each allele *a* on 70% of the typed individuals from the merged cohort (training set) by maximizing the log-likelihood, or equivalently by minimizing the logistic cross-entropy loss, with an L1 penalty which weight was optimized to minimize the loss function on the remaining 30% of individuals (testing set).

## Supporting information

S1 Fig Number of TCRs contributing to the determination of each loci. Number of specific CD4+ / CD8+ TCRs found to be specific to each loci. For both chains, the main CD4 and CD8 contributions are to the DQ and B loci determination, respectively. A residual (and likely false positive) association is found between CD4 TCRs and B loci.
(TIFF)

S2 Fig Additional parameters to describe HLA predictor performance. (A) Precision-recall curve of the classifier for the A*02:01 allele in the validation data set. (B) Area under the curve (AUC) of the ROC for each of the tested alleles. The average value of each group is indicated with the horizontal black line.
(TIFF)

S3 Fig Weights vs. significance comparison. (A) For each of the associations found for HLA A*02:01, the weight assigned by the logistic regression algorithm with L2 penalty (y-axis) is plotted against the significance of the TCR-HLA correlation, i.e. the p-value after Benjamini-Hochberg correction (x-axis). These p-values and weights exhibits a remarkable inverse correlation (Pearson −0.62, p-value: 3 × 10^−28^), confirming the idea that significantly HLA-associated TCRs are more helpful to determine the MHC type of the individual. (B) The use of L1 penalty promotes sparsity in the dataset, with most weights having zero value. The correlation between weight and significance is weaker in this case (Pearson coefficient: −0.30, p-value:3 × 10^−5^).
(TIFF)

S4 Fig HLA-related TCRs per age range. From *Britanova et al.* [41] the relative number of HLA-associated TCRs found in every individual is plotted against its age. Despite the suspicion that repertoires from older people may contribute with a higher number of associations due to successive antigen exposure and HLA-epitope restricton driven selection across lifetime, not a clear correlation is found (Pearson correlation coefficient: 0.29, p-value: 0.01).
(TIFF)

S1 Table Table of HLA-associated TCRs for both alpha and beta chain, containing their CDR3 amino acid sequence, V gene family, the number of individuals containing the given TCR and the subset of them that also are positive for the corresponding HLA allele, the corrected p-value (*p*_BH_) that characterizes their association as well as the kind of this last one (if there is a significant overexpression or underexpression of a TCR for the given HLA).
(XLSX)

S2 Table Summary of classifier parameters. Comparison of all classification metrics depending on the use of TCR*β* chain alone or together with TCR*α* chain.
(XLSX)

S3 Table Prediction comparison. Summary of the results (F1 score (A) and AUC (B)) reported in Akerman et al. 2023 [24] and DeWitt et al. 2018 [12] for the prediction of HLA-A and HLA-B alleles using only TCR*β* chain. Instead of the 4 digit resolution for the allele representation used in this work (e.g. A*02:01), these works made use of only two digits (e.g. A*02) leading to a better precision in prior works. HLAGuessr provides an overall improved performance by AUC of the ROC curve.
(XLSX)

## References

[1] Mora T, Walczak AM. How many different clonotypes do immune repertoires contain? Current Opinion in Systems Biology. 2019;18:104–110. doi: 10.1016/j.coisb.2019.10.001

[2] Garrett TPJ, Saper MA, Bjorkman PJ, Strominger JL, Wiley DC. Specificity pockets for the side chains of peptide antigens in HLA-Aw68. Nature. 1989;342(6250):692–696. doi: 10.1038/342692a0 2594067

[3] Marrack P, Scott-Browne JP, Dai S, Gapin L, Kappler JW. Evolutionarily conserved amino acids that control TCR-MHC interaction. Annual Review of Immunology. 2008;26(1):171–203. doi: 10.1146/annurev.immunol.26.021607.090421 18304006 PMC3164820

[4] Jurtz V, Paul S, Andreatta M, Marcatili P, Peters B, Nielsen M. NetMHCpan-4.0: Improved peptide–MHC Class I interaction predictions integrating eluted ligand and peptide binding affinity data. The Journal of Immunology. 2017;199(9):3360–3368. doi: 10.4049/jimmunol.1700893 28978689 PMC5679736

[5] Davis MM, Bjorkman PJ. T cell antigen receptor genes and T cell recognition. Nature. 1988;334(6181):395–402. doi: 10.1038/334395a0 3043226

[6] Krogsgaard M, Davis MM. How T cells “see” antigen. Nature Immunology. 2005;6(3):239–245. doi: 10.1038/ni1173 15716973

[7] Zhang H, Zhan X, Li B. GIANA allows computationally-efficient TCR clustering and multi-disease repertoire classification by isometric transformation. Nature Communications. 2021;12(1):4699. doi: 10.1038/s41467-021-25006-7 34349111 PMC8339063

[8] Sidhom JW, Larman HB, Pardoll DM, Baras AS. DeepTCR is a deep learning framework for revealing sequence concepts within T-cell repertoires. Nature Communications. 2021;12(1):1605. doi: 10.1038/s41467-021-21879-w 33707415 PMC7952906

[9] Liu M, Goo J, Liu Y, Sun W, Wu MC, Hsu L, et al. TCR-L: an analysis tool for evaluating the association between the T-cell receptor repertoire and clinical phenotypes. BMC Bioinformatics. 2022;23(1):152. doi: 10.1186/s12859-022-04690-2 35484495 PMC9052542

[10] Widrich M, Schäfl B, Pavlović M, Ramsauer H, Gruber L, Holzleitner M, et al. Modern Hopfield Networks and Attention for Immune Repertoire Classification. bioRxiv. 2020.

[11] Benichou J, Ben-Hamo R, Louzoun Y, Efroni S. Rep-Seq: uncovering the immunological repertoire through next-generation sequencing. Immunology. 2012;135(3):183–191. doi: 10.1111/j.1365-2567.2011.03527.x 22043864 PMC3311040

[12] DeWitt I William S, Smith A, Schoch G, Hansen JA, Matsen I Frederick A, Bradley P. Human T cell receptor occurrence patterns encode immune history, genetic background, and receptor specificity. eLife. 2018;7:e38358. doi: 10.7554/eLife.38358 30152754 PMC6162092

[13] Madi A, Shifrut E, Reich-Zeliger S, Gal H, Best K, Ndifon W, et al. T cell receptor repertoires share a restricted set of public and abundant CDR3 sequences that are associated with self-related immunity. Genome research. 2014;24 10:1603–12. doi: 10.1101/gr.170753.113 25024161 PMC4199372

[14] Madi A, Poran A, Shifrut E, Reich-Zeliger S, Greenstein E, Zaretsky I, et al. T cell receptor repertoires of mice and humans are clustered in similarity networks around conserved public CDR3 sequences. eLife. 2017;6:e22057. doi: 10.7554/eLife.22057 28731407 PMC5553937

[15] Venturi V, Kedzierska K, Price DA, Doherty PC, Douek DC, Turner SJ, et al. Sharing of T cell receptors in antigen-specific responses is driven by convergent recombination. Proceedings of the National Academy of Sciences. 2006;103(49):18691–18696. doi: 10.1073/pnas.0608907103 17130450 PMC1693724

[16] Venturi V, Price DA, Douek DC, Davenport MP. The molecular basis for public T cell responses. Nature Reviews Immunology. 2008;8(3):231–238. doi: 10.1038/nri2260 18301425

[17] Quigley MF, Greenaway HY, Venturi V, Lindsay R, Quinn KM, Seder RA, et al. Convergent recombination shapes the clonotypic landscape of the naïve T cell repertoire. Proceedings of the National Academy of Sciences. 2010;107(45):19414–19419. doi: 10.1073/pnas.1010586107 20974936 PMC2984183

[18] Murugan A, Mora T, Walczak AM, Callan CG. Statistical inference of the generation probability of T-cell receptors from sequence repertoires. Proceedings of the National Academy of Sciences of the United States of America. 2012;109(40):16161–6. doi: 10.1073/pnas.1212755109 22988065 PMC3479580

[19] Elhanati Y, Sethna Z, Callan CG Jr, Mora T, Walczak AM. Predicting the spectrum of TCR repertoire sharing with a data-driven model of recombination. Immunological Reviews. 2018;284(1):167–179. doi: 10.1111/imr.12665 29944757 PMC6033145

[20] Ruiz Ortega M, Spisak N, Mora T, Walczak AM. Modeling and predicting the overlap of B- and T-cell receptor repertoires in healthy and SARS-CoV-2 infected individuals. PLOS Genetics. 2023;19(2):1–29. doi: 10.1371/journal.pgen.1010652 36827454 PMC10075420

[21] Emerson RO, DeWitt WS, Vignali M, Gravley J, Hu JK, Osborne EJ, et al. Immunosequencing identifies signatures of cytomegalovirus exposure history and HLA-mediated effects on the T cell repertoire. Nature Genetics. 2017;49(5):659–665. doi: 10.1038/ng.3822 28369038

[22] May DH, Woodhouse S, Zahid HJ, Elyanow R, Doroschak K, Noakes MT, et al. Identifying immune signatures of common exposures through co-occurrence of T-cell receptors in tens of thousands of donors. bioRxiv. 2024.

[23] Zahid HJ, Taniguchi R, Ebert P, Chow IT, Gooley C, Lv J, et al. Large-scale statistical mapping of T-cell receptor *β* sequences to Human Leukocyte Antigens. bioRxiv. 2024. doi: 10.1101/2024.11.22.624922 39651287 PMC11623520

[24] Akerman O, Isakov H, Levi R, Psevkin V, Louzoun Y. Counting is almost all you need. Frontiers in Immunology. 2023;13. doi: 10.3389/fimmu.2022.1031011 36741395 PMC9896581

[25] Russell ML, Souquette A, Levine DM, Schattgen SA, Allen EK, Kuan G, et al. Combining genotypes and T cell receptor distributions to infer genetic loci determining V(D)J recombination probabilities. eLife. 2022;11:e73475. doi: 10.7554/eLife.73475 35315770 PMC8940181

[26] Rosati E, Martini GR, Pogorelyy MV, Minervina AA, Degenhardt F, Wendorff M, et al. A novel unconventional T cell population enriched in Crohn’s disease. Gut. 2022;71(11):2194–2204. doi: 10.1136/gutjnl-2021-325373 35264446 PMC9554086

[27] Alberts B, Johnson A, Lewis J, Raff M, Roberts K, Walter P. Molecular biology of the cell. Biochemistry and Molecular Biology Education. 2003;31(4):212–214.

[28] Milighetti M, Peng Y, Tan C, Mark M, Nageswaran G, Byrne S, et al. Large clones of pre-existing T cells drive early immunity against SARS-CoV-2 and LCMV infection. iScience. 2023;26:106937. doi: 10.1016/j.isci.2023.106937 37275518 PMC10201888

[29] The problem with neoantigen prediction. Nature Biotechnology. 2017;35(2):97–97. doi: 10.1038/nbt.3800 28178261

[30] Bassani-Sternberg M, Gfeller D. Unsupervised HLA peptidome deconvolution improves ligand prediction accuracy and predicts cooperative effects in peptide–HLA interactions. The Journal of Immunology. 2016;197(6):2492–2499. doi: 10.4049/jimmunol.1600808 27511729

[31] Bravi B, Tubiana J, Cocco S, Monasson R, Mora T, Walczak AM. RBM-MHC: A semi-supervised machine-learning method for sample-specific prediction of antigen presentation by HLA-I alleles. Cell Systems. 2021;12(2):195–202.e9. doi: 10.1016/j.cels.2020.11.005 33338400 PMC7895905

[32] Pogorelyy MV, Minervina AA, Shugay M, Chudakov DM, Lebedev YB, Mora T, et al. Detecting T cell receptors involved in immune responses from single repertoire snapshots. PLOS Biology. 2019;17(6):1–13. doi: 10.1371/journal.pbio.3000314 31194732 PMC6592544

[33] Dash P, Fiore-Gartland AJ, Hertz T, Wang GC, Sharma S, Souquette A, et al. Quantifiable predictive features define epitope-specific T cell receptor repertoires. Nature. 2017;547(7661):89–93. doi: 10.1038/nature22383 28636592 PMC5616171

[34] Venturi V, Quigley MF, Greenaway HY, Ng PC, Ende ZS, McIntosh T, et al. A mechanism for TCR sharing between T cell subsets and individuals revealed by pyrosequencing. The Journal of Immunology. 2011;186(7):4285–4294. doi: 10.4049/jimmunol.1003898 21383244

[35] Qi Q, Cavanagh MM, Le Saux S, NamKoong H, Kim C, Turgano E, et al. Diversification of the antigen-specific T cell receptor repertoire after varicella zoster vaccination. Science translational medicine. 2016;8(332):332ra46–332ra46. doi: 10.1126/scitranslmed.aaf1725 27030598 PMC4878824

[36] Varani L, Bankovich AJ, Liu CW, Colf LA, Jones LL, Kranz DM, et al. Solution mapping of T cell receptor docking footprints on peptide-MHC. Proceedings of the National Academy of Sciences. 2007;104(32):13080–13085. doi: 10.1073/pnas.0703702104 17670943 PMC1941830

[37] Bagaev DV, Vroomans RMA, Samir J, Stervbo U, Rius C, Dolton G, et al. VDJdb in 2019: Database Extension, New Analysis Infrastructure and a T-cell Receptor Motif Compendium. Nucleic Acids Research. 2020;48(D1):D1057–D1062. doi: 10.1093/nar/gkz874 31588507 PMC6943061

[38] 10x Genomics; 2020. https://pages.10xgenomics.com/rs/446-PBO-704/images/10x_AN047_IP_A_New_Way_of_Exploring_Immunity_Digital.pdf.

[39] Taub DD, Longo DL. Insights into thymic aging and regeneration. Immunological Reviews. 2005;205(1):72–93. doi: 10.1111/j.0105-2896.2005.00275.x 15882346

[40] Yager EJ, Ahmed M, Lanzer K, Randall TD, Woodland DL, Blackman MA. Age-associated decline in T cell repertoire diversity leads to holes in the repertoire and impaired immunity to influenza virus. Journal of Experimental Medicine. 2008;205(3):711–723. doi: 10.1084/jem.20071140 18332179 PMC2275391

[41] Britanova OV, Shugay M, Merzlyak EM, Staroverov DB, Putintseva EV, Turchaninova MA, et al. Dynamics of individual T cell repertoires: from cord Blood to centenarians. The Journal of Immunology. 2016;196(12):5005–5013. doi: 10.4049/jimmunol.1600005 27183615

[42] Nolan S, Vignali M, Klinger M, Dines JN, Kaplan IM, Svejnoha E, et al. A large-scale database of T cell receptor beta sequences and binding associations from natural and synthetic exposure to SARS-CoV-2. Research Square. 2020;. doi: 10.21203/rs.3.rs-51964/v1 32793896 PMC7418738

[43] Reynisson B, Alvarez B, Paul S, Peters B, Nielsen M. NetMHCpan-4.1 and NetMHCIIpan-4.0: Improved Predictions of MHC Antigen Presentation by Concurrent Motif Deconvolution and Integration of MS MHC Eluted Ligand Data. Nucleic Acids Research. 2020;48(W1):W449–W454. doi: 10.1093/nar/gkaa379 32406916 PMC7319546

[44] Montemurro A, Schuster V, Povlsen HR, Bentzen AK, Jurtz V, Chronister WD, et al. NetTCR-2.0 Enables Accurate Prediction of TCR-peptide Binding by Using Paired TCR*α* and *β* Sequence Data. Communications Biology. 2021;4(1):1–13. doi: 10.1038/s42003-021-02610-3 34508155 PMC8433451

[45] Meysman P, Barton J, Bravi B, Cohen-Lavi L, Karnaukhov V, Lilleskov E, et al. Benchmarking Solutions to the T-cell Receptor Epitope Prediction Problem: IMMREP22 Workshop Report. ImmunoInformatics. 2023;9:100024. doi: 10.1016/j.immuno.2023.100024

[46] Meynard-Piganeau B, Feinauer C, Weigt M, Walczak A, Mora T. TULIP—a Transformer Based Unsupervised Language Model for Interacting Peptides and T-cell Receptors That Generalizes to Unseen Epitopes. bioRxiv. 2023; p. 2023.07.19.549669. 10.1073/pnas.2316401121 PMC11181096 38838016

[47] Glazer N, Akerman O, Louzoun Y. Naive and Memory T Cells TCR–HLA-binding Prediction. Oxford Open Immunology. 2022;3(1):iqac001. doi: 10.1093/oxfimm/iqac001 36846560 PMC9914496

[48] Liu S, Bradley P, Sun W. Neural Network Models for Sequence-Based TCR and HLA Association Prediction. PLOS Computational Biology. 2023;19(11):e1011664. doi: 10.1371/journal.pcbi.1011664 37983288 PMC10695368

[49] Chronister WD, Crinklaw A, Mahajan S, Vita R, Koşaloğlu-Yalçın Z, Yan Z, et al. TCRMatch: Predicting T-cell receptor specificity based on sequence similarity to previously characterized receptors. Frontiers in Immunology. 2021;12. doi: 10.3389/fimmu.2021.640725 33777034 PMC7991084

[50] Springer I, Besser H, Tickotsky-Moskovitz N, Dvorkin S, Louzoun Y. Prediction of specific TCR-peptide binding from large dictionaries of TCR-peptide pairs. Frontiers in Immunology. 2020;11. doi: 10.3389/fimmu.2020.01803 32983088 PMC7477042

[51] Bolotin DA, Poslavsky S, Mitrophanov I, Shugay M, Mamedov IZ, Putintseva EV, et al. MiXCR: software for comprehensive adaptive immunity profiling. Nature Methods. 2015;12(5):380–381. doi: 10.1038/nmeth.3364 25924071

