# Supplementary figures and images for: Learning predictive signatures of HLA type from T-cell repertoires
Source: PLoS Comput Biol. 2025 Jan 6;21(1):e1012724. doi: 10.1371/journal.pcbi.1012724 (PMC11737854; doi:10.1371/journal.pcbi.1012724)

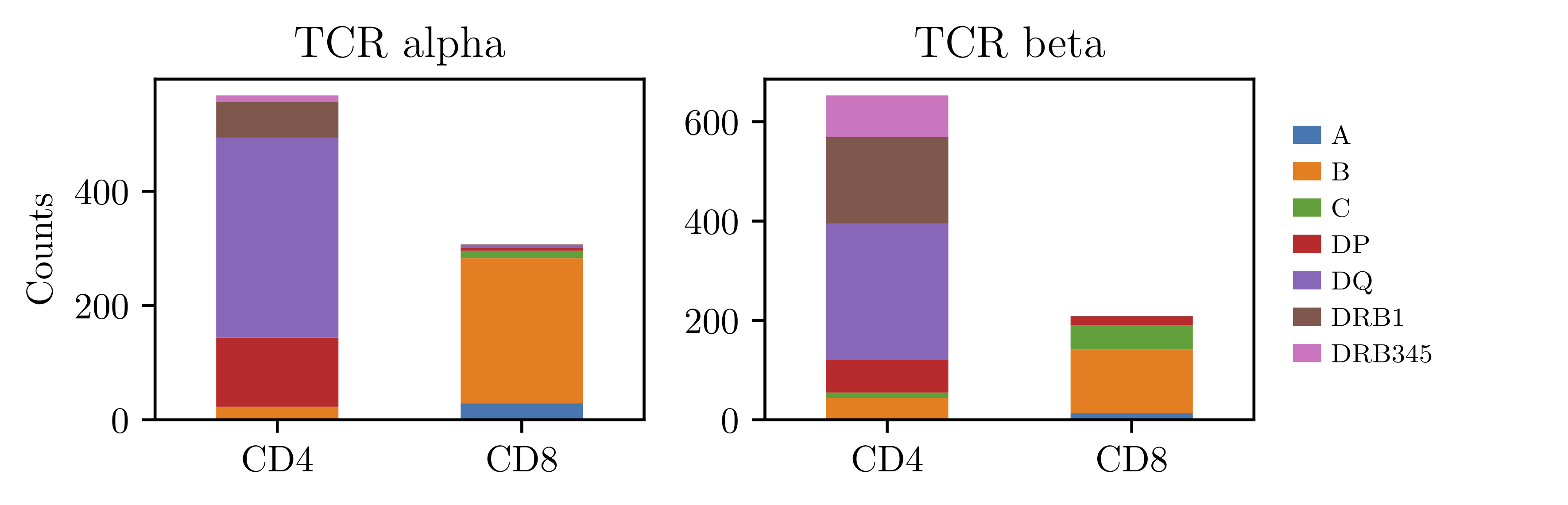

Supplement: S1 Fig — Number of specific CD4+ / CD8+ TCRs found to be specific to each loci. For both chains, the main CD4 and CD8 contributions are to the DQ and B loci determination, respectively. A residual (and likely false positive) association is found between CD4 TCRs and B loci. (TIFF) [file pcbi.1012724.s001.tiff]

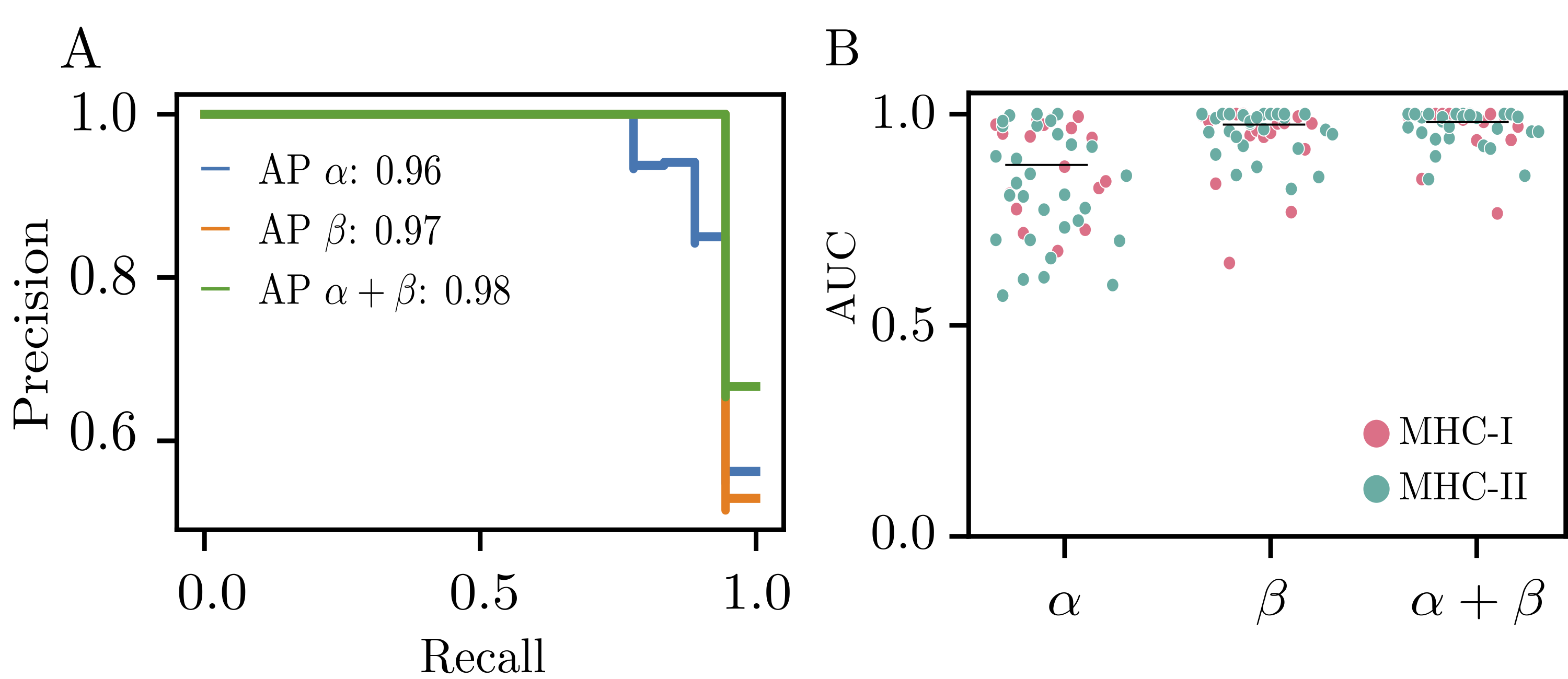

Supplement: S2 Fig — (A) Precision-recall curve of the classifier for the A*02:01 allele in the validation data set. (B) Area under the curve (AUC) of the ROC for each of the tested alleles. The average value of each group is indicated with the horizontal black line. (TIFF) [file pcbi.1012724.s002.tiff]

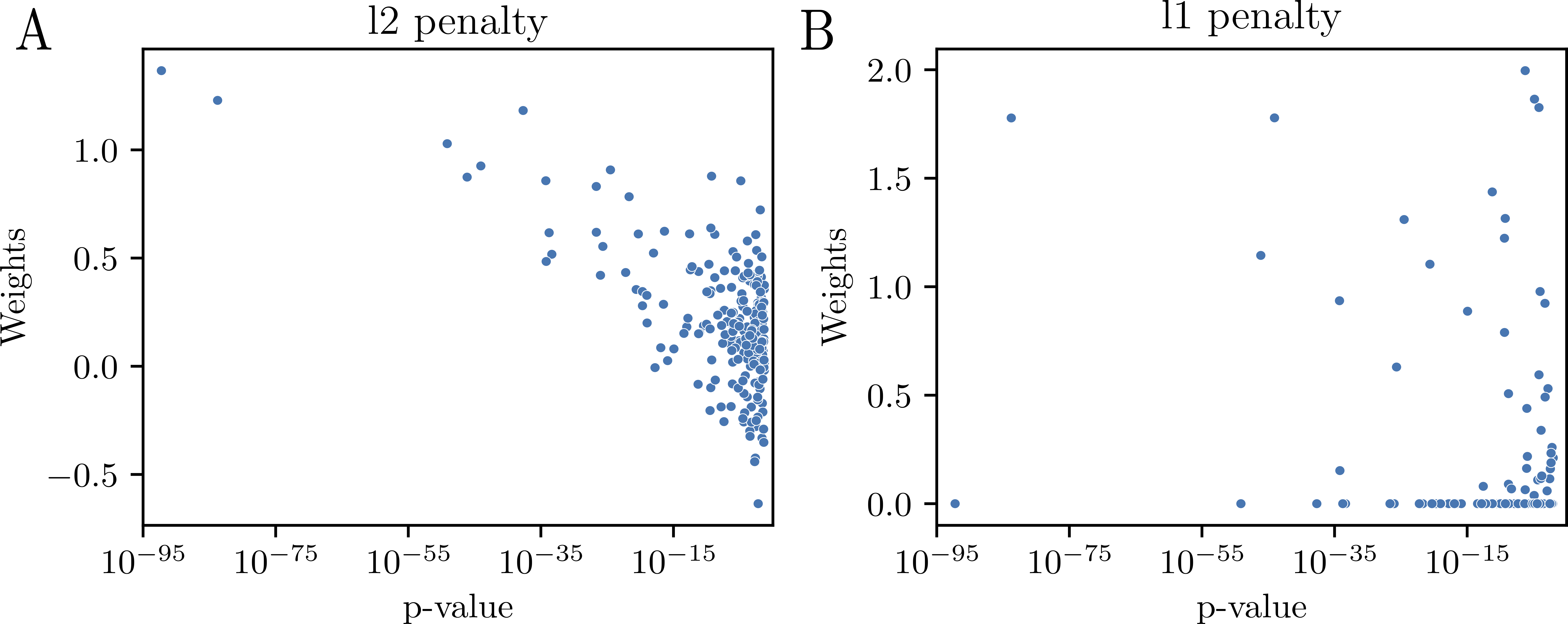

Supplement: S3 Fig — (A) For each of the associations found for HLA A*02:01, the weight assigned by the logistic regression algorithm with L2 penalty (y-axis) is plotted against the significance of the TCR-HLA correlation, i.e. the p-value after Benjamini-Hochberg correction (x-axis). These p-values and weights exhibits a remarkable inverse correlation (Pearson −0.62, p-value: 3 × 10^−28^), confirming the idea that significantly HLA-associated TCRs are more helpful to determine the MHC type of the individual. (B) The use of L1 penalty promotes sparsity in the dataset, with most weights having zero value. The correlation between weight and significance is weaker in this case (Pearson coefficient: −0.30, p-value:3 × 10^−5^). (TIFF) [file pcbi.1012724.s003.tiff]

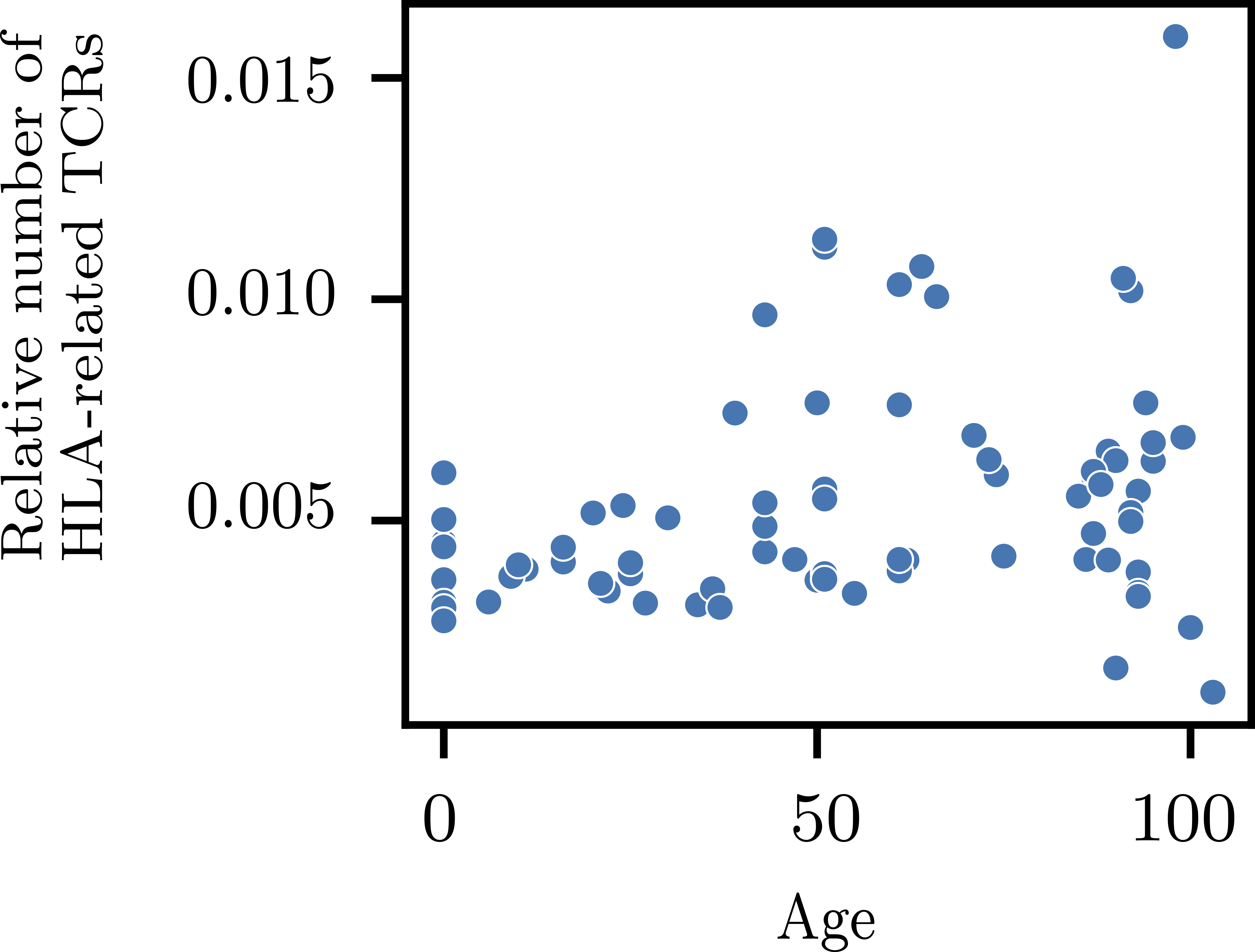

Supplement: S4 Fig — From Britanova et al. [41] the relative number of HLA-associated TCRs found in every individual is plotted against its age. Despite the suspicion that repertoires from older people may contribute with a higher number of associations due to successive antigen exposure and HLA-epitope restricton driven selection across lifetime, not a clear correlation is found (Pearson correlation coefficient: 0.29, p-value: 0.01). (TIFF) [file pcbi.1012724.s004.tiff]
